# Self-Control, Parental Monitoring, and Adolescent Problematic Mobile Phone Use: Testing the Interactive Effect and Its Gender Differences

**DOI:** 10.3389/fpsyg.2022.846618

**Published:** 2022-04-27

**Authors:** Yu-ting Hu, Qing Wang

**Affiliations:** ^1^School of Business, Jiangnan University, Wuxi, China; ^2^School of Humanities and Arts, China University of Mining and Technology, Xuzhou, China

**Keywords:** self-control, parental monitoring, gender differences, adolescents, problematic mobile phone use

## Abstract

Previous studies have revealed that self-control was one of the critical factors of adolescent problematic mobile phone use. Few studies, however, have explored the interaction of internal control force (i.e., self-control) and external control force such as parental monitoring. The present study tested the interactive effect of self-control and parental monitoring on adolescent problematic mobile phone use and its gender differences. A sample of 926 adolescents completed our anonymous self-report survey. Results showed that self-control and parental monitoring negatively predicted problematic mobile phone use, while gender positively predicted problematic mobile phone use. Self-control and parental monitoring had an interactive effect on problematic mobile phone use, with the effect of self-control on problematic mobile phone use being stronger for adolescents with low levels of parental monitoring than for those with high levels of parental monitoring. Self-control and gender had an interactive effect on problematic mobile phone use, with the effect of self-control on adolescent problematic mobile phone use being stronger in girls than in boys. Moreover, there were significant gender differences in the interaction of self-control and parental monitoring, in that the interactive effect of self-control and parental monitoring on problematic mobile phone use was more potent in girls than in boys. The findings reveal how the internal control force (i.e., self-control) and external control force (i.e., parental monitoring) work together in explaining adolescent problematic mobile phone use and uncover the potential gender differences in exploring adolescent problematic mobile phone use from the individual-environment perspective. Limitations and implications are discussed.

## Introduction

Problematic mobile phone use, also known as mobile phone addiction or mobile phone dependence, is a comprehensive state in which the physical and mental functions are obviously damaged due to the excessive use of mobile phones (Choliz, 2012; Lopez-Fernandez, 2017; Liu, 2019). Problematic mobile phone use has become a typical problem behavior for adolescent development in the mobile Internet era, with significant adverse effects on adolescents’ academic performance (Hawi and Samaha, 2016; Bai et al., 2020), social relationships (Seo et al., 2016), and physical and mental health (Goswami and Singh, 2016; Park et al., 2019; Hao et al., 2020). Individual factors are key proximal factors in adolescent problematic mobile phone use, of which self-control is a significant predictor of adolescent problematic mobile phone use (Khang et al., 2012). Self-control means that individuals actively control their emotions and behaviors for long-term goals (Tangney et al., 2004; Baumeister et al., 2007). It is a personality trait that plays an extremely important role in individual development (Duckworth, 2011; De Ridder et al., 2012). According to the self-regulation hypothesis of addiction, addictive behavior is essentially an individual’s lack of self-control and inability to suppress the excessive craving for the behavior (Kopetz et al., 2013; Gökçearslan et al., 2016). Many empirical studies have also found that self-control is a key predictor of adolescent problematic mobile phone use (Han et al., 2017; Liu et al., 2018b).

Although the relationship between self-control and adolescent problematic mobile phone use is supported theoretically and empirically, few studies have analyzed whether this relationship is influenced by protective environmental factors. According to the organism–environment interaction model (Lerner et al., 2006; Li et al., 2013), an individual’s development is influenced by both individual and environmental factors, with an interaction between the two. The effects of individual factors may also manifest differently in individuals with different environmental factors; likewise, the influence of environmental factors may manifest differently in individuals with different personality traits (Lerner et al., 2006). Parental monitoring, a family factor with protective effects, has a positive inhibitory effect on adolescent addictive behavior (Chang et al., 2019; Fu et al., 2020). Analyzing the interaction between self-control as an internal control force and parental monitoring as an external control force on adolescent problematic mobile phone use may reveal the predictive role of the individual–environment interaction on adolescent problematic mobile phone use in-depth and thus provide prevention and intervention measures for it as well as empirical support and scientific suggestions.

### Interactive Effects of Self-Control and Parental Monitoring

Parental monitoring refers to the monitoring of children’s behavior through parental attention, guidance, or discipline (Wang et al., 2007). Even in adolescents who are increasingly demanding autonomy, moderate parental control is an essential condition for their healthy development (Guilamo-Ramos et al., 2010a). According to social control theory (Hirschi, 1969), although humans have an innate urge to engage in many problematic behaviors, most people do not engage in transgressive behavior because there are various control mechanisms in society, such as family supervision and schooling. Parental monitoring is a direct and effective form of social control (Oxford et al., 2001; Ding et al., 2017). Studies have shown that parental monitoring not only directly reduces problem behaviors such as aggression, antisocial behavior, and substance abuse in adolescents (Skinner et al., 2014; Crocetti et al., 2016; Rusby et al., 2018) but also reduces the probability of problematic behaviors in adolescents by limiting their association with delinquent peers (Tompsett and Toro, 2010; van Ryzin et al., 2012).

The relationship between parental monitoring and multiple addictive behaviors has been confirmed by studies such as Strunin et al. (2013) who found that low levels of parental monitoring are significantly and positively associated with risky alcohol use among high school and college students. Lee et al. (2014) also confirmed that lower levels of parental monitoring in 11–14-year-old adolescents were significantly positively associated with gambling addiction in adulthood. Additionally, Ding et al. (2017) showed that parental monitoring is a significant and negative predictor of adolescent Internet addiction and that deviant peer affiliation played a mediating role in this relationship. Mobile phones are integrated into every aspect of people’s daily life in the mobile Internet era. Compared with the attention and vigilance regarding children’s excessive use of computers in the past, parents are increasingly accepting of adolescents’ mobile phone use; moreover, many parents place their children in electronic media environments with mobile phones and tablets at an early age. Thus, the lack of parental monitoring may be a more serious risk for adolescent problematic mobile phone use than has been shown in previous studies of Internet addiction. Empirical studies have also found a direct and significant predictive effect of parental monitoring on problematic mobile phone use among children and adolescents (Chang et al., 2019; Fu et al., 2020).

Self-control reflects the active regulation of behavior by internal forces, while parental monitoring reflects the supervision and guidance of behavior by external forces, and both types of control play a crucial role in individual development. Researchers argue that the current concern should not be about which is the more important role of the two, but about how they can work together to maximize their effects (Chen et al., 2015). According to the enhancing interaction hypothesis (Cohen et al., 2003; Liu et al., 2018b), the effect of one protective factor may be increased by another. Parental monitoring and self-control, two protective factors with positive effects, may also be enhanced by each other when they interact. That is, parental monitoring may enhance the predictive effect of self-control on adolescent problematic mobile phone use, and self-control may have a stronger predictive effect on adolescent problematic mobile phone use when the level of parental monitoring is high. Few studies, however, have confirmed the enhancing interaction hypothesis of the interaction between parenting and self-control. Nevertheless, the compensatory interaction hypothesis has been tested by some research. Previous studies have found an compensatory interaction between parental monitoring and self-control in some aspects of individual development, such as a significant reduction in antisocial behavior when there is more parental supervision for low self-control adolescents (Kuhn and Laird, 2013). In addition, parental presence and monitoring also help temperamentally impulsive and undercontrolled children to make more careful judgments, thereby reducing the risk of harm to the child (Schwebel and Bounds, 2003). The above studies suggest that the positive effects of parental supervision are stronger for individuals with low levels of self-control, meaning that parental supervision compensates for the negative developmental effects of low self-control. However, whether the interaction between parental monitoring and self-control on adolescent addictive behavior reinforces or compensates for each other has not been specifically explored in studies. Therefore, we put forward the hypothesis that parental monitoring will have a stronger effect on problematic mobile phone use in adolescents with lower levels of self-control (or self-control will have a stronger effect on problematic mobile phone use in adolescents with lower levels of parental monitoring).

### Gender Differences

In addition, there may be gender differences in the predictive effects of self-control and parental monitoring on adolescent problematic mobile phone use. Previous studies revealed significant gender differences in self-control, with males often having higher self-control than females and being better able to regulate mental resources to suppress various impulses (Shoenberger and Rocheleau, 2017; Wang et al., 2017); there may be significant gender differences in parental monitoring, with parents being more concerned about girls’ developmental issues, and thus, girls are more likely to be monitored by parents than boys (Guilamo-Ramos et al., 2010b). Significant gender differences in problematic mobile phone use have also been found, with females not only using mobile phones more frequently but also having more severe levels of addiction than males (Lee and Lee, 2017; Park et al., 2019; Liu et al., 2020). That is, girls already have more pronounced problematic mobile phone use problems than boys, and also face more risk factors for problematic mobile phone use; thus, the association between low self-control (or low parental monitoring) and problematic mobile phone use may be stronger among girls. At this point, high parental monitoring (or high self-control), as a positive factor, may provide a more significant mitigating effect? That is, high parental monitoring may exert a stronger buffering role in the association between low self-control and problematic mobile phone use in girls than in boys, and high self-control may exert a stronger buffering role in the association between low parental monitoring and problematic mobile phone use in girls than in boys. Therefore, we put forward the hypothesis that the interactive effect of parental monitoring and self-control on problematic mobile phone use will be stronger in girls than in boys.

### The Present Study

Parental monitoring, self-control, and gender are all vital predictors of problematic mobile phone addiction. However, no study to date has tested the interaction of parental monitoring and self-control on adolescent problematic mobile phone use and its gender differences. This study focused on adolescent problematic mobile phone use, examining whether self-control and parental monitoring interact to predict adolescent problematic mobile phone use from the perspective of the individual–environment interaction; it further analyzed whether there are gender differences in this interaction. The present study would generate significant theoretical and practical implications.

## Materials and Methods

### Participants and Procedure

The research has been approved by the Ethical Committee for Scientific Research at the first author’s institution. Two middle schools and two high schools were selected as the target schools. The cluster random sampling method was used to choose two classes in each grade from grade 7 to grade 9 in middle schools, and two classes in each grade from grade 10 to 12 in high schools. The informed consent was obtained from all the participants and the schools. Well-trained psychology students acted as the investigators to conduct the anonymous survey in classrooms during normal school lessons. A total of 926 adolescents completed the survey to provide information regarding self-control, parental monitoring, problematic mobile phone use, and demographic variables. These participants were between 12 and 18 years of age, and the mean age was 14.53 years old (SD*_*age*_* = 1.788). Among these participants, there were 472 (50.97%) middle school students and 454 (49.07%) high school students, and 496 (53.56%) boys and 430 (46.44%) girls.

### Measurements

#### Self-Control

Self-control was measured by the Chinese version of Self-control Scale developed by Dong and Lin (2011). Participants rated five items on a four-point scale (1 = strongly disagree, 4 = strongly agree). Higher scores reflect better self-control. Cronbach’s α for this scale in our study was 0.87. The Chinese version of the Self-control Scale has shown good validity and reliability in adolescents in previous studies (e.g., Liu et al., 2018a; Zheng et al., 2019).

#### Parental Monitoring

Parental monitoring was assessed by the Chinese version (Liu, 2019) of the Strictness/Supervision Scale (Steinberg et al., 1992). This scale consists of four items (e.g., “how much do your parents try to know where you go at night”) rated on a five-point (0 = never, 4 = always). Higher scores indicate stronger parental monitoring. Cronbach’s α coefficient for this scale was 0.77. The Strictness/Supervision Scale has shown reasonable validity and reliability in Chinese adolescents in previous research (e.g., Liu, 2019; Bi et al., 2020).

#### Problematic Mobile Phone Use

Problematic mobile phone use was measured by the Chinese version (Cheung et al., 2019) of Smartphone Addiction Scale-Short Version (Kwon et al., 2013). Participants answered ten items (e.g., “I will never give up using my smartphone even when my daily life is already greatly affected by it”) on a five-point scale (1 = *never*, 5 = *always*). Higher scores indicate greater problematic mobile phone use. Cronbach’s α coefficient for this scale in our study was 0.92. The Chinese version of the Smartphone Addiction Scale-Short Version has shown good validity and reliability in previous studies (e.g., Cheung et al., 2019; Shen et al., 2022).

### Statistical Analyses

Descriptive statistics and Pearson correlation analyses were first conducted using SPSS 23.0. Then we analyzed a three-way interaction model to reveal the interaction of self-control and parental monitoring on adolescent problematic mobile phone use and its gender differences using the PROCESS macro for SPSS (Hayes, 2013). We included age as the control variable in the interaction model analysis due to the potential age differences in self-control and problematic mobile phone use (van Deursen et al., 2015; Wang et al., 2017). In addition, we also included daily mobile phone use time as a covariate in the model test because previous research found that mobile phone use time was positively associated with problematic mobile phone use (Kuss et al., 2018).

## Results

### Preliminary Analyses

There were significant gender differences in the scores of self-control, parental monitoring, and problematic mobile phone use. Specifically, boys scored higher on self-control than girls (*t* = 6.72, *p* < 0.001), while girls scored higher on parental monitoring (*t* = 6.81, *p* < 0.001) and problematic mobile phone use than boys (*t* = 7.29, *p* < 0.001). The descriptive statistics and correlation matrix are presented in Table 1. In both boy and girl groups, self-control was positively correlated with parental monitoring, while self-control and parental monitoring were both negatively correlated with problematic mobile phone use.

**TABLE 1:** Descriptive statistics and correlations between variables.

Variables	*M*(SD) for boys	*M*(SD) for girls	1	2	3
1. Self-control	2.69(0.69)	2.32(0.86)	–	0.34***	−0.45***
2. Parental monitoring	2.64(1.04)	2.93(0.82)	0.28***	–	−0.36***
3. Problematic mobile phone use	2.17(0.83)	2.65(1.14)	−0.36***	−0.27***	–

N = 926. Values below and above the diagonal represent boy and girl sample, respectively. ***p < 0.001.

### Testing for the Interaction Model

The interaction of self-control and parental monitoring on adolescent problematic mobile phone use and its gender differences is a question of a three-way interaction between self-control, parental monitoring, and gender. We conducted a three-way interaction analysis using the PROCESS macro for SPSS (Hayes, 2013). Table 2 presents the regression results of the three-way interactive model.

**TABLE 2:** Three-way interaction model analysis.

Regression equation		Significance of regression coefficients				Model fit index	
Outcome	Independent variables	B	*SE*	*t*	*p*	*R* ^2^	*F*
Problematic mobile phone use	Constant	−0.09**	0.03	–2.97	<0.01	0.31	41.31***
	Age	0.005	0.03	0.17	0.86		
	Daily mobile phone use time	0.03	0.03	1.12	0.26		
	Self-control	−0.35***	0.03	–10.37	<0.001		
	Parental monitoring	−0.12**	0.04	–3.22	<0.01		
	Gender	0.28***	0.06	4.84	<0.001		
	Self-control × Parental monitoring	0.24***	0.04	5.59	<0.001		
	Self-control × Gender	−0.22***	0.07	–3.32	<0.001		
	Parental monitoring × Gender	0.07	0.08	0.92	0.36		
	Three-way interaction	0.27**	0.09	3.10	<0.01		

N = 926. Bootstrap sample size = 5,000. SE, standard error. Three-way interaction = self-control × parental monitoring × gender. **p < 0.001. ***p < 0.001.

**FIGURE 1:**
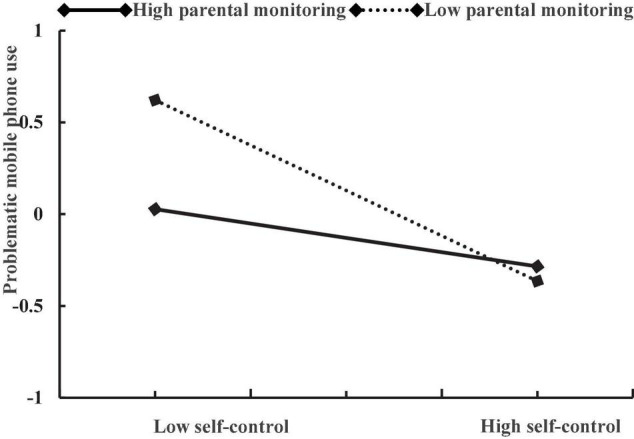
The relationship between self-control and problematic mobile phone use at two levels of parental monitoring: (1) low parental monitoring (1 SD below the mean) and (2) high parental monitoring (1 SD above the mean).

**FIGURE 2:**
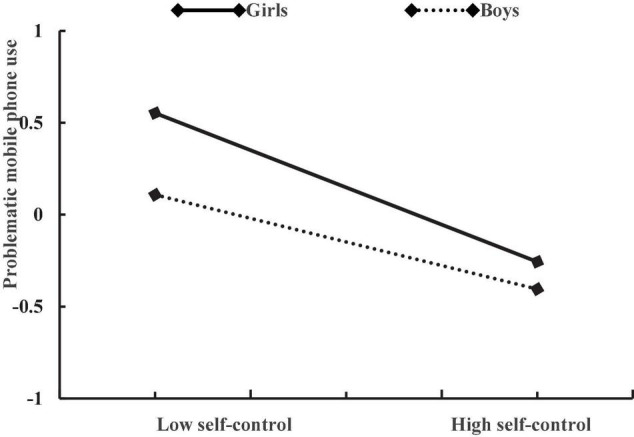
The relationship between self-control and problematic mobile phone use in boys and girls.

After controlling for age and daily mobile phone use time, self-control negatively predicted problematic mobile phone use (β = −0.35, *p* < 0.001), parental monitoring negatively predicted problematic mobile phone use (β = −0.12, *p* < 0.01), and gender positively predicted problematic mobile phone use (β = 0.28, *p* < 0.001). The interaction of self-control and parental monitoring had a positively effect on adolescent problematic mobile phone use (β = 0.24, *p* < 0.001), the interaction of self-control and gender also had a positively effect on adolescent problematic mobile phone use (β = −0.22, *p* < 0.001), while the interactive effect of parental monitoring and gender on adolescent problematic mobile phone use was not significant (β = 0.07, *p* = 0.36). The plot of the association between self-control and adolescent problematic mobile phone use at two values of parental monitoring (1 SD below the mean and 1 SD above the mean) is described in Figure 1. The association between self-control and problematic mobile phone use was stronger for adolescents with low levels of parental monitoring (β = −0.60, *p* < 0.001) than for those with high levels of parental monitoring (β = −0.17, *p* < 0.01). The plot of the association between self-control and adolescent problematic mobile phone use among boys and girls is described in Figure 2. The association between self-control and problematic mobile phone use was stronger in girls (β = −0.49, *p* < 0.001) than in boys (β = −0.34, *p* < 0.001).

**FIGURE 3:**
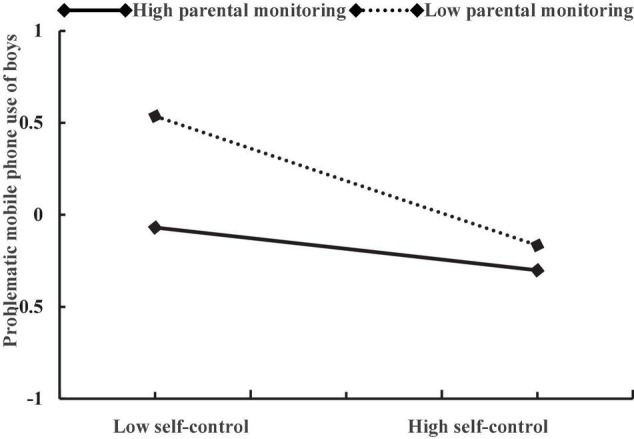
The relationship between between self-control and problematic mobile phone use of boys at two levels of parental monitoring: (1) low parental monitoring (1 SD below the mean) and (2) high parental monitoring (1 SD above the mean).

**FIGURE 4:**
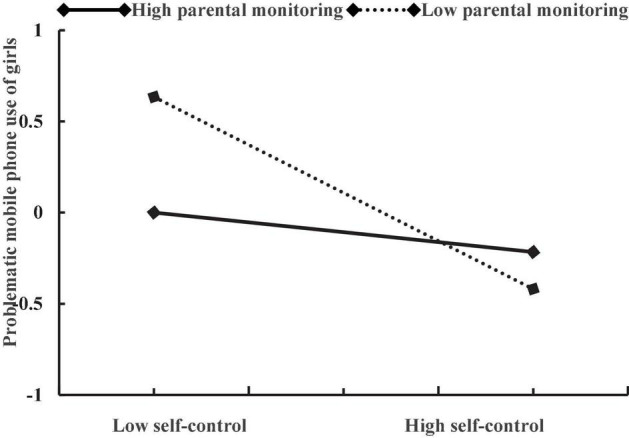
The relationship between between self-control and problematic mobile phone use of girls at two levels of parental monitoring: (1) low parental monitoring (1 SD below the mean) and (2) high parental monitoring (1 SD above the mean).

Moreover, the three-way interaction of self-control, parental monitoring, and gender also had a positive effect on problematic mobile phone use (β = 0.27, *p* < 0.01). As shown in Table 3, in girls, the interactive effect of self-control and parental monitoring on adolescent problematic mobile phone use was strong (β = 0.38, *p* < 0.001). In boys, although the interactive effect of self-control and parental monitoring on adolescent problematic mobile phone use was still significant, it is relatively weak (β = 0.11, *p* < 0.05). Specifically (see Table 4), in boys, the association between self-control and problematic mobile phone use was stronger for adolescents with low levels of parental monitoring (β =−0.44, *p* < 0.001) than for those with high levels of parental monitoring (β =−0.15, *p* < 0.05). In girls, self-control was positively associated with problematic mobile phone use (β =−0.67, *p* < 0.001) for adolescents with low levels of parental monitoring, but this association became non-significant for adolescents with high levels of parental monitoring (β =−0.07, *p* = 0.38). The plots of the association between self-control and adolescent problematic mobile phone use at two values of parental monitoring (1 SD below the mean and 1 SD above the mean) among boys and girls are described in Figures 3, 4, respectively.

**TABLE 3:** Conditional interactive effects of self-control and parental monitoring in boys and girls.

Group	Coefficient	*SE*	*t*	*p*	Bootstrap LLCI	Bootstrap ULCI
Boys	0.11***	0.05	2.20	<0.05	0.01	0.22
Girls	0.38**	0.07	5.47	<0.001	0.25	0.52

N = 926. Bootstrap sample size = 5,000. SE, standard error; LL, low limit; CI, confidence interval; UL, upper limit. **p < 0.01. ***p < 0.001.

**TABLE 4:** Effects of self-control on problematic mobile phone use at values of parental monitoring in boys and girls, respectively.

Group	Values of parental monitoring	Coefficient	*SE*	*t*	*p*	Bootstrap LLCI	Bootstrap ULCI
Boys	M - SD	−0.44***	0.09	–4.83	<0.001	–0.62	–0.26
	M	−0.30***	0.05	–5.56	<0.001	–0.40	–0.19
	M + SD	−0.15*	0.08	–1.99	<0.05	–0.31	–0.002
Girls	M - SD	−0.67***	0.06	–11.45	<0.001	–0.78	–0.56
	M	−0.37***	0.05	–7.84	<0.001	–0.46	–0.28
	M + SD	–0.07	0.08	–0.89	0.38	–0.24	0.09

N = 926. Bootstrap sample size = 5,000. SE, standard error; LL, low limit; CI, confidence interval, UL, upper limit. *p < 0.05; ***p < 0.001.

In sum, the results indicated that self-control and parental monitoring had a significant interactive effect on adolescent problematic mobile phone use and this interactive effect showed significant gender differences.

## Discussion

With the development of the mobile Internet, adolescent problematic mobile phone use has increasingly become a significant issue of social concern. External environmental factors and internal individual factors are two fundamental causes of adolescent problematic mobile phone use. To the best of our knowledge, few studies to date have tested the interaction of external and internal control forces on adolescent problematic mobile phone use and its gender differences. This study explored the interaction between self-control and parental monitoring on adolescents’ problematic mobile phone use from the perspective of individual–environment interaction and further examined whether there were gender differences in the interaction. The results found that the interaction between self-control and parental monitoring was a significant predictor of adolescent problematic mobile phone use and that the two factors show a compensatory interaction pattern; the predictive effect of parental monitoring on adolescent problematic mobile phone use was stronger in adolescents with low levels of self-control than for those with high levels of self-control. Conversely, the predictive effect of self-control on adolescent problematic mobile phone use was stronger in adolescents with low levels of parental monitoring than for those with high levels of parental monitoring. In addition, there were significant gender differences in the interactive effect of self-control and parental monitoring on adolescent problematic mobile phone use, with the interaction between self-control and parental monitoring being stronger in girls than boys. From the perspective of theoretical innovation, the present study would help expand the validity of the organism-environment interaction model and the complementary interaction model since these theories were applied to support the hypothesis of the interaction between parental monitoring and self-control, and the results confirmed the effects of external and internal control forces on adolescent problematic mobile phone use. Our study may also encourage more researchers to explore adolescent problematic mobile phone use from the individual-environment interaction perspective and the complementary interaction model. From the perspective of practical innovation, the present study would provide practical guides for the prevention and intervention of problematic mobile phone use among adolescents with low parental monitoring and those with low self-control from the pathway of combining the internal and external control forces. The practical implications may be particularly effective because they take internal and external control forces and gender differences into account.

The present study found that the interaction between self-control and parental monitoring predicted adolescent problematic mobile phone use, which is consistent with the organism–environment interaction model (Lerner et al., 2006) and the compensatory interaction model (Cohen et al., 2003; Liu et al., 2018b). According to the organism–environment interaction model (Lerner et al., 2006), individual and environmental factors do not independently affect people’s physical and mental development. They exert impacts on individuals in an interactive way. On this basis, the complementary interaction hypothesis further describes the specific model of interaction. That is, internal control forces and external control forces complement each other, and they can alleviate the adverse effects caused by the lack of each other. Previous research has found that the indirect effect of school connectedness on adolescent Internet addiction was moderated by self-control, with the indirect effect being stronger for adolescents with low self-control (Li et al., 2013). Although the complementary interaction hypothesis has been tested in research on adolescent Internet addiction, few studies have examined the interaction between parental monitoring (as the external control force) and self-control (as the internal control force) on adolescent problematic mobile phone use. The results of the present study suggest that the forces of internal and external control compensate for each other in adolescent problematic mobile phone use, with high self-control positively compensating for the negative effects of low parental monitoring on adolescent problematic mobile phone use and high parental monitoring positively compensating for the negative effects of low self-control on adolescent problematic mobile phone use. Self-control is a crucial proximal factor of adolescent problematic mobile phone use (Khang et al., 2012), and adolescents with high self-control can better control their excessive craving for mobile phones cognitively and their excessive use of mobile phones behaviorally, so that they can effectively regulate their behavior and avoid problematic mobile phone use even without parental monitoring. Similarly, parental monitoring is an important external factor of adolescent problematic mobile phone use (Fu et al., 2020), and parents can effectively limit adolescents’ mobile phone use time and guide adolescents’ mobile phone use behavior. Hence, even adolescents with low self-control may use mobile phones reasonably under parental monitoring. The results of this study suggest that both self-control as an internal control force and parental monitoring as an external control force have critical impacts on adolescent behavior as they not only independently predict adolescent problematic mobile phone use but also work synergistically in a mutually compensatory manner to predict it. The results also emphasize that the roles of self-control and parental monitoring may be equally important in adolescent addictive behaviors and that their interaction can be considered to better describe and predict adolescent addictive behaviors.

This study also found a significant gender difference in the interaction between self-control and parental control on adolescent problematic mobile phone use. The interaction between self-control and parental control on adolescent problematic mobile phone use was more significant among girls than boys. That is, girls with low parental monitoring benefited more from self-control than boys with low parental monitoring; girls with low self-control benefited more from parental monitoring than boys with low self-control; increasing levels of self-control tended to keep girls (but not boys) with deficient parental monitoring away from problematic mobile phone use; and higher parental monitoring kept girls (but not boys) with deficient self-control more away from problematic mobile phone use. Many previous studies have found that girls often have less self-control than boys (Shoenberger and Rocheleau, 2017; Wang et al., 2017), making them more likely to become addicted when confronted with a mobile phone (Park et al., 2019; Liu et al., 2020). That is, the link between self-control and problematic mobile phone use may be stronger among girls compared to boys. As parental monitoring has an important effect on adolescent problematic mobile phone use and can positively compensate for the negative effects of inadequate self-control, parental monitoring is more likely to mitigate the predictive effect of inadequate self-control on adolescent problematic mobile phone use in girls compared to boys. Similarly, self-control was more likely to mitigate the negative effects of a lack of parental monitoring on adolescent problematic mobile phone use in girls. The results of this study suggest that although girls have more severe problems with problematic mobile phone use than boys, these addiction problems can be greatly improved by reinforcing either positive individual (e.g., self-control) or environmental (e.g., parental monitoring) factors. Studies on the factors influencing problematic mobile phone use and the effects of interventions should focus more on the female population.

## Limitations and Implications

There are several limitations to this study. First, our study adopted a cross-sectional questionnaire design and could not rigorously determine the direct effects of self-control and parental monitoring on adolescent problematic mobile phone use. A longitudinal questionnaire design could be employed in future studies to examine the causal relationship. Second, our study used a subjective reporting method to collect data, which may be subject to social approval effects and memory bias, and future studies could consider multiple sources (data from self and others) and methods (subjective reports and objective responses) for data collection. Finally, although we conducted the study from an individual–environment interaction perspective, we only analyzed the interaction of individual factors with family factors and did not address other environmental factors (e.g., school or peer environments). Future studies could consider analyzing the interaction between individual factors and school or peer factors on adolescent problematic mobile phone use.

Despite several shortcomings, the present study still has essential theoretical and practical implications. In the theoretical sense, our study constructed an integrated model to explore the interaction between self-control and parental monitoring on adolescent problematic mobile phone use and its gender differences, and the findings conform to the organism–environment interaction model to verify the interaction between individual and environmental factors on adolescent problematic mobile phone use and its gender differences. The results can help expand existing research on the influencing factors of adolescent problematic mobile phone use, enrich the research results on these influencing factors, and reveal the independent and joint roles of different influencing factors. In practical terms, this study can encourage more researchers to analyze the development of adolescent problematic mobile phone use from the perspective of individual–environment interaction, such as exploring the interaction between more individual and family factors or comprehensively examining the complex interaction between individual, family, school, and peer factors. Our study can also provide empirical support and practical recommendations for the prevention and intervention of adolescent problematic mobile phone use, such as considering the role of both external and internal control forces to decrease the risk of problematic mobile phone use in adolescents with inadequate self-control through enhanced parental monitoring, and conversely, to decrease the risk of problematic mobile phone use in adolescents with absent parental monitoring through enhanced self-control. Additionally, increasing self-control or parental monitoring would be particularly useful in alleviating problematic mobile phone use among females.

## Conclusion

In conclusion, we conducted a three-way interaction model to analyze the interactive effect of self-control and parental monitoring on adolescent problematic mobile phone use and its gender differences. Self-control and parental monitoring had an interactive effect on problematic mobile phone use, in that the effect of self-control on problematic mobile phone use was stronger for adolescents with lower levels of parental monitoring, and the effect of parental monitoring on problematic mobile phone use was stronger for adolescents with lower levels of self-control. Moreover, there were significant gender differences in the interaction of self-control and parental monitoring, with the interactive effect of self-control and parental monitoring on problematic mobile phone use being stronger in girls than in boys. The findings highlight the interaction of the internal control force (i.e., self-control) and external control force (i.e., parental monitoring) and its gender differences in exploring adolescent problematic mobile phone use from the individual-environment perspective.

## Data Availability Statement

The raw data supporting the conclusions of this article will be made available by the authors, without undue reservation.

## Ethics Statement

The studies involving human participants were reviewed and approved by Academic Committee of Business School of Jiangnan University. Written informed consent to participate in this study was provided by the participants’ legal guardian/next of kin.

## Author Contributions

Y-TH was responsible for research design and manuscript writing. QW reviewed the manuscript. Both authors contributed to the article and approved the submitted version.

## Conflict of Interest

The authors declare that the research was conducted in the absence of any commercial or financial relationships that could be construed as a potential conflict of interest.

## Publisher’s Note

All claims expressed in this article are solely those of the authors and do not necessarily represent those of their affiliated organizations, or those of the publisher, the editors and the reviewers. Any product that may be evaluated in this article, or claim that may be made by its manufacturer, is not guaranteed or endorsed by the publisher.
